# Noninvasive Measurement of Infant Respiration During Sleep: A Validation Study

**DOI:** 10.3390/s26072275

**Published:** 2026-04-07

**Authors:** Melissa N. Horger, Maristella Lucchini, Shambhavi Thakur, Rebecca M. C. Spencer, Natalie Barnett

**Affiliations:** 1 Department of Psychological and Brain Sciences, College of Natural Sciences, University of Massachusetts Amherst, Amherst, MA 01003, USA; rspencer@umass.edu; 2 Nanit Lab, New York, NY 10001, USA; maristella.lucchini@nanit.com (M.L.); shambhavi.thakur@nanit.com (S.T.); natalie.barnett@nanit.com (N.B.)

**Keywords:** breathing, in home, methodology, nap, videosomnography

## Abstract

**Highlights:**

**What is the main finding?**

- Infant respiration was measured equivalently from cardiorespiratory sensors and the Nanit computer vision algorithms, which detected movement of the pattern printed on a cloth band worn around the torso (Nanit Breathing Band).

**What is the implication of the main finding?**

- Noninvasive measurement of respiration with commercial devices can improve infant sleep and health monitoring.

**Abstract:**

Infant respiration is a physiological marker of health and wellbeing that can provide insight into sleep and wake patterns. Technological innovation presents opportunities to enhance measurements of physiological signals, which improves ecological validity and participant experiences. This is particularly true in the context of studying infant sleep, as it can be disrupted by changes in the environment and the physical sensation of unfamiliar or uncomfortable sensors. The goal of this study was to examine if a commercially available video baby monitor (Nanit system) can accurately estimate respiration during a nap relative to a commonly used cardiorespiratory sensor (Isansys Lifetouch sensor). Thirty-three infants (M = 9.7 months; range = 1–22 months) took a nap while wearing the Lifetouch sensor and Nanit Breathing Band. Infants slept in view of the Nanit camera. A computer vision algorithm applied to the video detected movement of the patterns on the fabric band worn around the infant’s torso to determine respiratory rates. The results showed strong consistency between the devices. More than 95% of the minute-by-minute respiration data fell within the limits of agreement, with little bias. Agreement was not influenced by age or nap duration, suggesting the Nanit Breathing Band provides a valid measure of respiration across infancy.

## 1. Introduction

Infant respiration is of critical interest to pediatricians, researchers, clinicians, and caregivers. Cardiorespiratory functioning is regularly monitored in typical populations and more closely tracked in premature infants, who commonly require high levels of pulmonary support. Respiration is linked to both immediate and long-term health and neurodevelopmental outcomes [1].

Respiration is measured in a variety of ways such as through sound detection, plethysmography, a pulse oximeter, ECG monitoring, radar, and thermal imaging. Tradeoffs exist in accuracy and invasiveness among methods, as well as in their appropriateness for long-term monitoring [2,3,4]. Some of these techniques have been adopted for non-clinical use and commercial sale [5]. Their ease of use allows commercial versions of these techniques to be applied by caregivers and become part of everyday routines. Such tools shed light on infants’ states and behaviors as they record and summarize data. One such application is in the field of infant sleep monitoring [6].

Measuring respiration can unlock real-time information about aspects of sleep, since this respiration changes not only from sleep to wake, but also in the transition from NREM to REM sleep, thus providing valuable insights into sleep quality, duration, and macrostructure. Resulting information may be valuable to caregivers, researchers and perhaps clinicians since sleep is a critical part of infancy, comprising most of an infant’s first year (>12 h/day at 3 months; >11 h/day at 12 months) [7,8]. Importantly, infant sleep has been linked to a variety of developmental outcomes such as cognition [7,9,10,11,12], neural development [13,14], and physical health [15]. Infant sleep also predicts parent wellbeing [16,17].

The goal of the current study was to explore the validity of a commercially available video-based baby monitoring system (Nanit system) and proprietary computer vision algorithm to quantify infant respiration during sleep. Respiratory rates derived from Nanit were compared to those from a wearable cardiorespiratory sensor which served as the gold standard.

## 2. Materials and Methods

### 2.1. Participants

Forty-two infants aged 1–22 months (M = 9.7 months; SD = 5.7 months) and their caregivers participated in the study. The caregivers received a free subscription to the Nanit Insights Ultimate tier in the app ($300 value) as compensation for their time. They were recruited between June and November 2024 through an email sent to Nanit users who provided their information when registering for the app. Interested families completed screening, and those meeting eligibility criteria were contacted via email to explain the procedure and, if interested, schedule their session around the infant’s usual nap time. Typically developing, healthy 1- to 24-month-old infants were included in the study. We focused on this age range because we were recruiting from Nanit users and the product was designed for infants and toddlers. Exclusion criteria included premature birth (< 34 weeks gestation), recent or chronic cardiorespiratory or dermatological issues, and a diagnosed developmental, sleep, or respiratory disorder. Demographic information was not collected but previous research with US-based Nanit app users reported predominantly White samples with an annual household income >$150,000 [18].

### 2.2. Measures of Respiration

**Figure 1:**
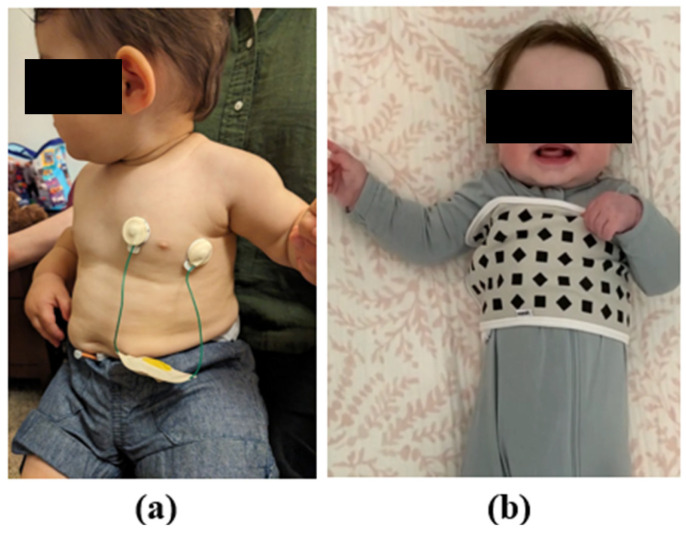
Visualization of the procedure including (**a**) the Lifetouch Blue sensor and (**b**) the Nanit Breathing Band.

Wearable, wireless cardiorespiratory sensors (Lifetouch Blue sensors, Isansys Lifecare Ltd., Oxford, UK) provided continuous measures for heart rate (beats per minute) and respiratory rate (breaths per minute), sampled at 1000 Hz and aggregated in 1-min increments [19]. Lifetouch Blue sensors are commonly used in hospital settings, including pediatric units [20,21,22], and served as the gold standard. They have been validated against direct observation in adults [23]. The sensors comprise 2 electrodes placed roughly on either side of the heart (see Figure 1a) and transmit data over Bluetooth to an AWS server. Data were then downloaded for the entire sleep period in 1-min epochs.

The Nanit Breathing Band is a cotton band printed with a high contrast geometric pattern which is affixed around the infant’s chest with a Velcro strip (see Figure 1b). The Nanit Breathing Band is detected by the Nanit camera (dba Udisense Inc., New York, NY, USA) which leverages a proprietary algorithm to measure movement of the geometric pattern corresponding to the rise and fall of the infant’s chest. The Nanit camera uses infrared LEDs to clearly capture black-and-white night vision videos in dark rooms. Data was stored securely on Amazon Web Services (AWS)’s Simple Storage Service (S3) and subsequently downloaded using AWS Command Line Interface (CLI) for the entire sleep period in 1-min epochs.

Both systems relied on tablet-based applications to begin the measurement window. The Lifetouch sensors were activated by the Patient Status Engine and the sleep interval onset was marked by the Nanit app. The tablets and subsequent data time stamps were synced to local time. Onboard, proprietary pre-processing from both devices generated the 1-min epochs which were then screened for artifacts generated by movement or technical issues. Movement artifacts were identified in the Isansys data as outliers with accompanying increases in activity measured by the device’s onboard accelerometer. Movement artifacts in the Nanit data were identified by epochs with <30 s within the 1 min epoch, providing usable data because the Breathing Band was occluded or unclear. Additional outliers (>3 SD above the mean) were removed from further analysis.

### 2.3. Procedure

IRB approval was obtained from the University of Massachusetts Amherst (Protocol #4678). Caregivers provided written informed consent online through REDCap, a survey platform, before beginning the study. The consent form outlined the potential risks of the study, including breach of confidentiality from video and discomfort during the application or removal of the sensors. Infants were given a unique identifier, under which all data was stored and saved to protect participant confidentiality. Sessions were scheduled around infants’ typical nap times. All naps occurred in a nursery set up in the Nanit offices (New York, NY), which was equipped with a crib, rocker, and infant-friendly decorations. The room was furnished with blackout curtains and the overhead lights were turned off once the infant was put down for their nap. No blankets were placed in the crib to avoid visual occlusion of the Breathing Band which is in alignment with instructions provided for commercial users. The Nanit camera was centered on the long side of the crib and positioned between it and the wall.

After parents provided informed consent, infants were outfitted with the Lifetouch sensor and the Nanit Breathing Band. Caregivers continued with their usual sleep routine and infants were allowed to sleep until naturally waking.

### 2.4. Data Analysis Plan

We estimated the necessary sample size with a mean difference of 0, standard deviation of 1.5, and maximal allowable difference score of 5. With these criteria, the necessary sample size (or number of observations) was 33 with 80% power [24]. We oversampled the total number of participants to account for collinearity within subjects’ data.

All analyses were conducted using RStudio (2025.05.1). Data manipulation was conducted using dplyr, tidyverse, hms, and lubridate packages. To assess the validity of the Nanit Breathing Band, we compared respiratory rates derived from the Nanit Breathing Band to respiratory rates derived from the Lifetouch Blue sensor using mean absolute error, repeated measures correlations, and Bland–Altman plots, modified for repeated measures [25]. The mean absolute error was calculated using the Metrics package. The predicted values were generated using the rnorm function with the distribution parameters used in the power analysis (M = 0, SD = 1.5). Repeated-measures correlations were calculated with the rmcorr package. Correlations quantified agreement between methods, while Bland–Altman plots captured systematic bias or random variation [26,27,28,29]

Bland–Altman plots depict breaths per minute (BPM) averaged across the two devices on the X-axis and the difference scores on the Y-axis. Difference scores were calculated as:

(1) $$d=BPM\left(Lifetouch\right)-BPM(Nanit\;Breathing\;Band)$$

The average distance was plotted as a horizontal line along with upper and lower limits of agreement (LOAs) which represent the confidence interval (1.96*SD_d_). To account for repeated measures within participants and data in which the true value varies at each measurement point, we calculated the LOA using the formula outlined in the prior literature [29,30]. The total variance (*σ*^2^*_T_*) was estimated in two parts: for repeated differences between the two methods on the same subject (*σ*^2^*_W_*) and for averages of the two methods across subjects (*σ*^2^*_B_*). A one-way ANOVA (*d* ~ participant) provided the within-subject mean square, estimating the variance within subjects (*MS_W_* = *σ*^2^*_W_*). Variance between the subjects (*σ*^2^*_B_*) was estimated using the following formula:

(2) $$\sigma_{B}^{2}=\left(MS_{B}-MS_{w}\right)∕\frac{{\left(\sum m_{i}\right)}^{2}+\Sigma_{m_{i}}^{2}}{\left(n-1\right)\sum m_{i}}$$

We have *m* epochs or pairs of observations for subject *i* and there were *n* subjects. The between-subjects mean square (*MS_B_*) was generated in the ANOVA along with the MS_W_. Finally, the standard deviation (*SD_d_*) was calculated as:

(3) $${SD}_{d}=\sqrt{\left(\sigma_{B}^{2}+\sigma_{W}^{2}\right)}$$

Resulting visualizations were plotted using the ggplot2 package.

## 3. Results

From our total sample, usable data were available from *n* = 33 participants (*M_age_* = 9.7 months, *SD* = 5.7, range = 1–22 months). Data from nine participants were removed from further analysis due to technical issues (*n* = 6) or inability to fall asleep (*n* = 3). Naps lasted an average of 57.9 min (*SD* = 40.1; range = 18–191 min). The resulting data were screened for motion artifacts and we rejected 16 epochs (0.83%) from the Isansys data, 72 (3.8%) from the Breathing Band data, and 18 (0.94%) from both. Fifty additional epochs (2.6%) were rejected from the Isansys data because they contained outliers (>3 SD + Mean).

### Comparison of Methods for Recording Respiration

**Figure 2:**
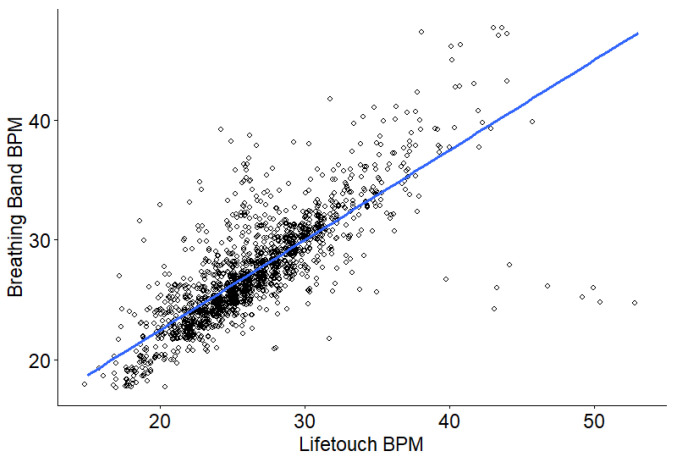
Scatterplot of breaths per minute reported by Lifetouch Blue sensor and Nanit Breathing Band.

**Figure 3:**
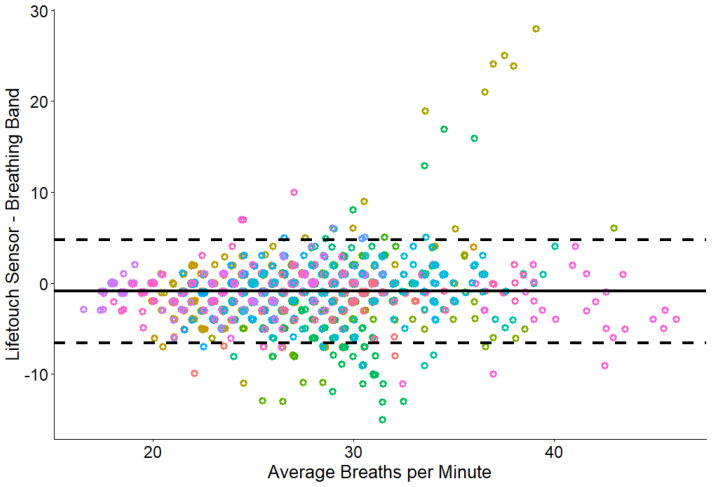
Bland–Altman plot comparing minute-by-minute respiratory rates recorded by the Lifetouch sensor and Nanit Breathing Band. Dashed lines represent the upper and lower LOAs [−6.58, 4.76]. The solid line represents the average difference score (*d_Mean_* = −0.91). Participant level data were signified by color as they had variable nap lengths and contributed unequally to the total sample.

We included 1755 total minutes or epochs (91.8%) of respiratory data in the analysis. The mean absolute difference between the two methods was 1.61 (CI: 1.52, 1.71). Respiratory rates derived from the Nanit Breathing Band were significantly correlated with those from the Lifetouch sensor (*r* = 0.47, *p* < 0.001, CI: 0.438, 0.512) (Figure 2). The Bland–Altman plot comparing the two measures (Figure 3) shows limits of agreement [−6.58, 4.76] around the average difference score (*M* = −0.91). A total of 95.3% of the data fell within these boundaries.

**Figure 4:**
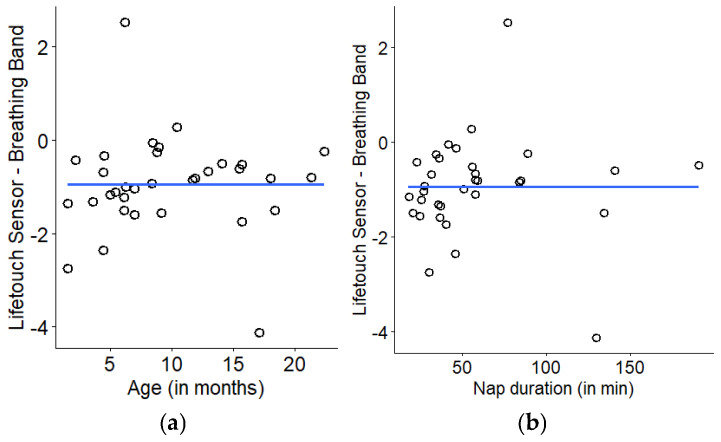
Scatterplots of each participants’ average difference score and (**a**) age and (**b**) nap duration.

Infants consistently napped longer with age (*r* = 0.6, *p* < 0.001). To examine potential changes in measurement accuracy due to age and recording duration, we assessed the impact of both on difference scores (d). Participants’ mean d values were unrelated to age (r = −0.004) and nap duration (r = 0.001) (Figure 4a and Figure 4b, respectively).

## 4. Discussion

The present study evaluated the accuracy of the Nanit Breathing Band and corresponding computer vision algorithm to derive respiratory rates during sleep in infants and toddlers relative to a common respiratory sensor. The mean absolute error was low (MAE = 1.61), repeated-measures correlation coefficients were moderate, and more than 95% of the respiration data fell within the Bland–Altman limits of agreement. Taken together, these results suggest that the Nanit system provides an accurate measure of infant respiration during sleep bouts.

When examining the Bland–Altman plot, the points that fall outside the limits of agreement further inform our understanding of how the two methods can differ. Overall, there is little bias between the methods as the average difference score (−0.91) was just below 0. All difference scores tended to fall equally above and below the average, and importantly, we did not see a consistent bias for increased error with higher respiratory rates. The outlying cluster of positive difference scores in the graph were attributable to two participants for unknown reasons. A recent assessment of contact-free respiratory monitoring in an NICU using a radar above the infant compared to traditional monitoring reported Bland–Altman plots with an average difference score of −0.296 and LOAs of 7.64 bpm and −8.24 bpm, which is very similar to our findings [31]. In older children (1–15 years old), a threshold of ±5 breaths per minute has been used as the “maximal width of the limits of agreement to not impair medical care” [28].

When difference scores were compared to age and nap duration, we found no relationship. The efficacy of the Nanit Breathing Band seems to be unaffected by age or recording duration. Such evidence bolsters the effectiveness of this measurement technique across the infant and toddler period when shorter sleep bouts consolidate into longer, but less frequent periods. It also demonstrates that the Nanit Breathing Band is sensitive enough to capture developmental fluctuations in respiration, as rates decrease with age.

There is potential that this new form of monitoring may be leveraged to monitor populations at elevated risk for sleep disordered breathing, allowing for early identification and data-driven treatment plans. However, more research is needed before transitioning to clinical research. Our upper limit of agreement was within the recommended interval for pediatric monitoring (±5) but our lower limit exceeded it, suggesting accuracy could yet be improved. Additionally, it is unknown how performance may change as a function of crib mattress or sleep environment, as all infants were tested in the exact same nursery. We also do not know how robust the computer vision algorithm is across sleep stages or to changes in body position. Only daytime naps were included in the present study, but overnight sleep would include more time spent in different sleep stages (NREM and REM) as well as more opportunities for movement. It is unclear how Nanit would compare to other noninvasive, commercial devices which are similarly impacted by factors like movement and body position. Ultimately, direct study of the use of this technology in clinical populations, such as those with respiratory pathology, is necessary as the current work only focused on healthy infants. Nanit’s Breathing Band and computer vision algorithm offer a scalable solution to the noted limitations of existing sleep measurement techniques in pediatric populations. While this commercial technology is most commonly used in the home by caregivers, it may be considered by researchers, particularly as a way to collect longitudinal and densely sampled data which is needed to capture intra- and interindividual differences in sleep patterns across early life. In this way, leveraging commercially available devices can mutually benefit caregivers and researchers. Families gain access to real-time, useful information (from the accompanying Nanit app in this instance) and researchers can collect naturalistic, longitudinal data with minimal participant burden.

## Data Availability

Data can be provided upon request. Accompanying code can be found online at https://github.com/mhorger/NanitBreathingBandValidationStudy/ (accessed on 26 March 2026).

## Abbreviation

The following abbreviation is used in this manuscript:

BPM	Breaths per Minute
